# Gene Ontology term overlap as a measure of gene functional similarity

**DOI:** 10.1186/1471-2105-9-327

**Published:** 2008-08-04

**Authors:** Meeta Mistry, Paul Pavlidis

**Affiliations:** 1 CIHR/MSFHR Graduate Program in Bioinformatics, University of British Columbia, Canada; 2 Department of Psychiatry and Centre for High-throughput Biology, University of British Columbia, British Columbia, Canada

## Abstract

**Background:**

The availability of various high-throughput experimental and computational methods allows biologists to rapidly infer functional relationships between genes. It is often necessary to evaluate these predictions computationally, a task that requires a reference database for functional relatedness. One such reference is the Gene Ontology (GO). A number of groups have suggested that the semantic similarity of the GO annotations of genes can serve as a proxy for functional relatedness. Here we evaluate a simple measure of semantic similarity, term overlap (TO).

**Results:**

We computed the TO for randomly selected gene pairs from the mouse genome. For comparison, we implemented six previously reported semantic similarity measures that share the feature of using computation of probabilities of terms to infer information content, in addition to three vector based approaches and a normalized version of the TO measure. We find that the overlap measure is highly correlated with the others but differs in detail. TO is at least as good a predictor of sequence similarity as the other measures. We further show that term overlap may avoid some problems that affect the probability-based measures. Term overlap is also much faster to compute than the information content-based measures.

**Conclusion:**

Our experiments suggest that term overlap can serve as a simple and fast alternative to other approaches which use explicit information content estimation or require complex pre-calculations, while also avoiding problems that some other measures may encounter.

## Background

In this paper we consider the problem of deciding if two genes are functionally related using computational methods. In particular, we are interested in how existing information about gene function can be used to enhance or evaluate computational predictions of functional relationships among genes.

Many genes have been functionally characterized by experimental methods, sequencing efforts, and high-throughput techniques, and as a consequence those genes then appear in public databases annotated with terms or concepts representative of their deduced function or biological role in the cell. The Gene Ontology (GO) is a structured, controlled vocabulary of terms providing consistency in annotating how a given gene product behaves in a cellular context, and many genes are now annotated with terms from GO [1]. It is increasingly common to attempt to define functional relatedness using "semantic similarity" of genes using GO annotations [2-7]. While many measures have been used, their relative benefits and drawbacks are unclear. The current work involves an examination of the behaviour of various semantic similarity measures that have been proposed, including one that has not been previously considered in comparisons.

**Figure 1:**
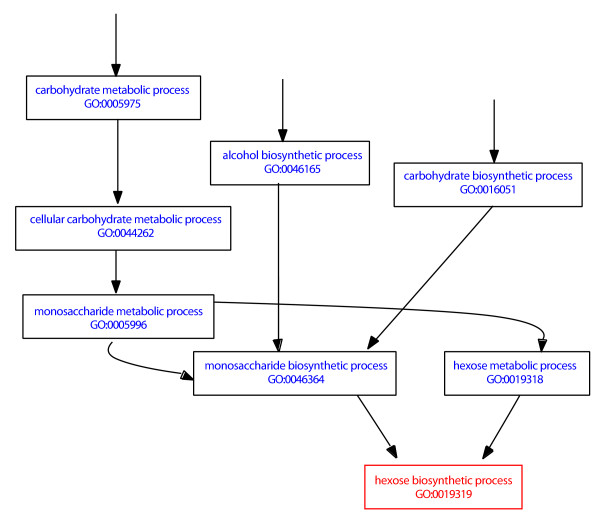
**Structure of Gene Ontology**. Depicted here is a graphical representation of the GO term "hexose biosynthetic process" and its associated parent terms, adapted from the AmiGO website . Only partial paths are shown here. The three paths branch up to higher level parent terms, leading back to the root term "biological process". Arrows between terms represent 'is-a' relationships. The hierarchy of each ontology is structured as a directed acyclic graph (DAG), with the more specific, lower level terms, having one or more direct parent terms associated with it. This is because a single child term can be defined in a number of different contexts.

Six of the measures we consider in this work make use of the hierarchical structure of the GO. Each term in GO is assigned to one of the three root ontologies: molecular function, biological process and cellular component. The terms in each ontology are linked to one another in an acyclic directed graph by two types of relationships: 'is-a', which represents a simple class-subclass relationship and 'part-of', which indicates a component relationship [8]. Importantly, terms can have more than one direct parent term, because a single child term can be defined in a number of different contexts. For example, the biological process term "hexose biosynthetic process" (GO:0019319) has two direct parent terms, "hexose metabolic process" and "monosaccharide biosynthetic process". This is because biosynthesis is a subtype of metabolism, and a hexose is also a type of monosaccharide (Figure 1). The GO terms used by genome database curators in the direct annotation of a gene are usually more specific, lower level terms. However, the graphical structure of the GO implies that a gene that is associated with a low level term is also associated with higher level terms. Thus a gene involved in "hexose metabolic process" can also be given the annotation "metabolic process" by inference.

Previous work on the use of the GO to measure functional similarity focussed on the use of information content [9,10]. The information content (IC) of a term is related to how often the term is applied to genes in the database, such that rarely used terms are ascribed higher IC. The IC for GO terms is monotonically decreasing as one follows the graph from a leaf terms towards the root term. Intuitively, terms low in the hierarchy are "more detailed" and impart more information about function than high-level terms such as "metabolism". Semantic similarity measures based on IC make use of the idea that genes sharing terms with high IC are expected to be more functionally similar than terms that share terms with low IC. Indeed, it was shown that some IC-based measures correlate with other measures of functional relatedness such as sequence similarity [9,10].

A second set of semantic similarity measures are variations on the Vector Space Model (VSM) [11], an algebraic model originally developed for use in information retrieval. Unlike the IC-based measures, these methods do not account for hierarchical relations in the GO, and instead refer to GO terms in a 'flat' matrix format. The requirement of individual gene vectors to be generated is an extra complexity cost that is incurred prior to the actual similarity computation itself. In this study we implement three of such methods [12-14] for comparison against our measure.

Our proposed method, Term Overlap (TO), was used previously by Lee et al. [4] in a study of gene coexpression analysis, where it was shown that TO correlates with increasing confidence in coexpression. Although Lee et al. [4] first implemented the TO measure, it was not thoroughly evaluated nor was it compared to other similarity measures. In this study we sought to test whether TO is an adequate substitute for other measures that have been put forward. In contrast to the other semantic similarity measures, TO does not use an explicit information content computation, and is less algorithmically complex. Here we explore the properties of TO in more detail and carry out a more formal evaluation of the approach, and find that TO has a number of attractive features that may recommend it as an alternative to other semantic similarity measures.

## Methods

### Data Sources

Sets of gene pairs were generated by random pairwise selection from the mouse genome. Each of the genes was annotated with its respective GO terms as it appears in the NCBI Gene database [15] (downloaded on January 8, 2008). Genes which were not annotated with any GO terms were not considered, leaving a set of 18,161 genes. Several sets containing 10,000 gene pairs each were evaluated initially, with a final dataset of 100,000 gene pairs generated for which the results are displayed in this paper ("100 k"). The 100 k set has pairs covering the entire corpus of mouse genes. The 100 k set with associated statistics is available as supplementary data from .

### Information Content and Semantic Similarity Measures

Several of the measures we considered require the computation of the information content of each GO term. These measures were originally described for the analysis of any corpus of text, and were adapted for use with GO by Lord et al. (2003), where full details are given. The information content of a GO term *t*_*i *_is:

(1) *IC*(*t*_*i*_) = -log(*p*(*t*_*i*_))

Where *p*(*t*_*i*_) is the probability of a term occurring in the corpus:

(2) *p*(*t*_*i*_) = *freq*(*t*_*i*_)/*freq*(*root*)

where the corpus is the set of annotations for all genes under consideration. "Root" represents one of the three root ontology terms and *freq*(*root*) is the number of times a gene is annotated with any term within that ontology. *freq*(*t*_*i*_) is given by:

(3) $$freq(t_{i})=\left|annot(t_{i})\right|+\sum_{c\in children(t_{i})}\left|annot(c)\right|$$

Where *children*(*t*_*i*_) is the set of all children terms for the term *t*_*i *_(that is, the set of all terms for which *t*_*i *_is a parent term, either directly or indirectly).

In our analysis we focus on three IC based measures adapted from the work of Resnik[16], Lin[17], Jiang and Conrath[18]. Resnik's measure calculates the similarity between two terms by using only the IC of the lowest common ancestor (LCA) shared between two terms *t*_1 _and *t*_2 _:

(4) *sim*_*Res*_(*t*_1_, *t*_2_) = *IC*(*LCA*)

Lin's measure of similarity takes into consideration the IC values for each of terms *t*_1 _and *t*_2 _in addition to the LCA shared between the two terms and is defined as follows [17]:

(5) $$sim_{Lin}(t_{1},t_{2})=\frac{2\log(p(LCA))}{\log p(t_{1})+\log p(t_{2})}$$

Jiang and Conrath proposed an IC based semantic distance, which can be transformed into a similarity measure [6]:

(6) $$sim_{Jiang}(t_{1},t_{2})=\frac{1}{-\log(p(t_{1}))-\log(p(t_{2}))+2\log(p(LCA))+1}$$

For each of the three measures, a higher score indicates a higher semantic similarity between two terms. The lowest score for all three measures is 0. The highest score for Lin and Jiang is 1, and Resnik's measure has no upper bound.

These measures are intended to score the similarity between two GO terms, and must be extended to compare genes, each of which can have multiple GO terms. Following the approach of [9], let us compare two gene products *g*_1 _and *g*_2_. Every term in the direct annotation set for gene *g*_1 _is compared against every term in the direct annotation set for gene *g*_2_. For each pairwise comparison if two direct annotations are identical, that term is then considered the LCA. If two direct annotations are not identical, we then retrieve the parent term sets induced for the two annotation terms, and the shared parent term with the highest information content is considered the LCA. The similarity score is then calculated for that pair of terms. The scores generated for all pairs of GO terms are used to produce a final score for the gene pair in one of two ways: i) scores can be averaged across all possible term pairs for the two genes [9] or ii) only the maximum score resulting from all possible term pairs for the two genes is used, as proposed by [19]. We refer to these as the "average" and "maximum" methods in the following. Thus we consider six IC-based measures: Li, Jiang and Resnik, each with average and maximum variants.

### Vector Space Model Measures

These similarity measures first require an *m *× *n *gene-term annotation matrix be compiled, where *m *is the total number of genes in the corpus and *n *is the total number of GO terms. Each row in the matrix represents a gene vector of its annotations. Each vector is binary valued, with 1 representing the presence of the GO term in the gene's annotation and 0 representing its absence. The Cosine similarity can be calculated using the vector for each gene in the pair [14].

(7) $$sim_{cos}(g_{1},g_{2})=\frac{v_{1}\cdot v_{2}}{\left|v_{1}\right|\left|v_{2}\right|}$$

A variation on the Cosine measure, which has been previously used in ontology-based similarity, first generates a weight, *w*_*t*_, for each GO term based on the frequency of its occurrence in the corpus [12].

(8) *w*_*t *_= log(*N*/*n*_*t*_)

Where *N *is the total number of genes in the corpus and *n*_*t *_is the number of genes in the corpus annotated with that term *t*. These weights replace the non-zero values in the binary vector and similarly the cosine measure is calculated as in (7). We refer to this method as the Weighted Cosine measure in this study.

Finally, Huang et al also propose a vector-based similarity measure integrated in the DAVID Gene Functional Classification Tool [13]. For a given gene pair, binary gene vectors are extracted from the compiled matrix as described above. Kappa statistics are then used to measure co-occurrence of annotation between gene pairs. The algorithm can be found in detail in [13].

### Term Overlap Measure

When calculating the term overlap between two gene products we consider the set of all direct annotations for each gene and all of their associated parent terms (excluding the root of the hierarchy) as a gene product annotation set, *annot*_*g*1_. The term overlap score for two genes is then calculated as the number of terms that occur in the intersection set of the two gene product annotation sets.

(9) *sim*_*TO*_(*g*_1_, *g*_2_) = |*annot*_*g*1 _∩ *annot*_*g*2_|

As with the other measures, the higher the score the higher the similarity between two genes. The lowest term overlap score is zero and there is no upper bound. The similarity of genes where one or both lacks GO terms is zero, though as mentioned these were not considered here. A variant method we also considered is the normalized term overlap (NTO), in which the term overlap score is divided by the annotation set size for the gene with the lower number of GO annotations.

(10) $$sim_{NTO}(g_{1},g_{2})=\frac{sim_{TO}(g_{1},g_{2})}{min(|annot_{g1}|,|annot_{g2}|)}$$

Traditional cardinality-based similarity measures such as Jaccard and Dice [14] are computed similarly to NTO, but use the union or sum, respectively, of the two gene annotation set sizes as the normalizing factor. The Czekanowski-Dice distance used in the functional analyses module of GOToolBox [20], calculates a distance by normalizing the number of symmetric differences between the two gene term sets with the sum of the intersection and union sets. In this way, the scale for the distance is reversed from that of NTO, with genes having no GO terms in common scoring a distance of 1 and highly functionally related scoring closer to 0. Since these measures are very similar to NTO, we chose not to include them in our study.

### Sequence analysis

The NCBI Consensus Coding Sequence (CCDS) protein sequences were obtained from the NCBI FTP site . We used the NCBI blast suite program "bl2seq" to analyze the similarity between the protein sequences for each of the 100,000 gene pair [21]. Similarity for this analysis was measured using the bit score values. CCDS did not include sequences for some of the genes considered, yielding similarity scores for 67,179 pairs. Correlations and their statistical significance were determined using the cor.test function in R [22].

## Results

For each data set (consisting of randomly selected pairs of mouse genes), we computed similarity scores using TO, NTO, each of the IC-based similarity measures (Resnik, Lin and Jiang, using both the "average" and "maximum" variants for each), and each of the three vector-based measures (Cosine, Weighted Cosine and Kappa) for a total of eleven measures. We then sought to see how well the scores generated from the different measures correlated with one another. A correlation analysis was repeated for several sets of 10,000 randomly chosen gene pairs. We found the variation between the results for each of the sets to be negligible (Additional file 1), thus only data from a final 100 K gene pair set was studied in detail.

**Figure 2:**
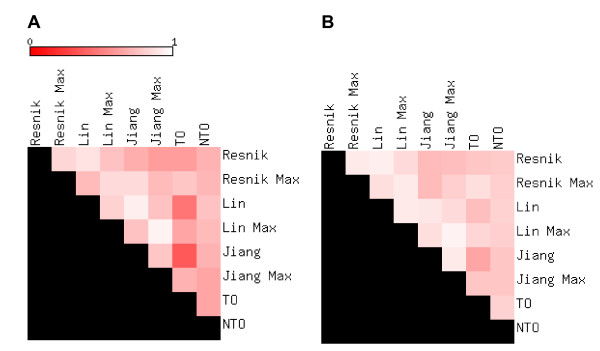
**Measuring correlation between similarity scores**. Pearson (A) and Spearman rank correlation (B) values were calculated to measure the degree of agreement between scores generated using each of the various measures. Scores were generated for the 100 k dataset for each method. Correlation was evaluated for scores between all possible pairs of measures.

**Figure 3:**
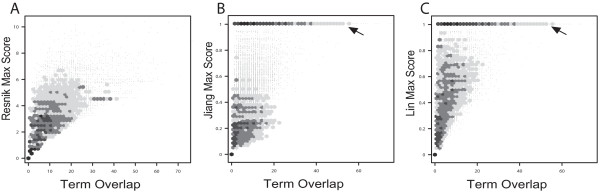
**Relationship between Term Overlap and alternate methods ("maximum" variants)**. The data for the 100 k set of randomly selected gene pairs are presented as raw points (light grey) as well as density (hexagons, plotted using the R package "hexbin"). Darker colors indicate increasing density of points. The plots represent TO vs. Resnik (A), Jiang (B), and Lin (C).

**Figure 4:**
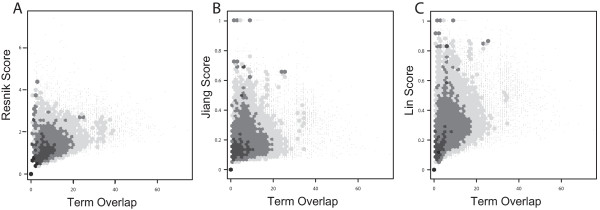
**Relationship between Term Overlap and alternate methods ("average" variants)**. The plots represent TO vs. Resnik (A), Jiang (B), and Lin (C). For details see figure 3.

**Figure 5:**
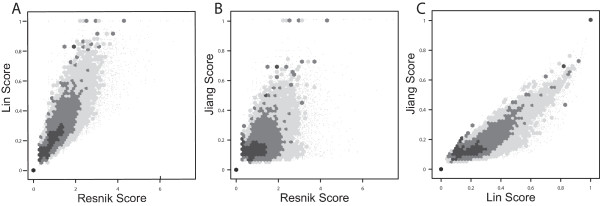
**Comparisons among information-content methods**. Similarity scores were calculated using the averaged variant of Resnik, Lin and Jiang similarity measures for every gene pair in the 100 k set of randomly selected gene pairs. The scores generated were then plotted against each other to illustrate the correlation amongst them, A) Resnik versus Lin; B) Resnik versus Jiang; C) Lin versus Jiang.

Figure 2A and 2B are heat-map representations of the Pearson and rank correlations, respectively, among each of the eleven measures. Some numerical data are shown in Table 1 (the full data set can be found in Additional file 2). Several trends are evident: all of the measures are positively correlated with TO, and furthermore the other measures are also correlated with each other. The relationship with TO and Resnik-maximum was amongst the strongest of all TO measure correlations and also relatively linear, giving high values of 0.87 and 0.77 for both rank and Pearson correlations respectively. Both Cosine and Kappa methods also showed strong correlations with TO, with slightly higher rank values of 0.89 and 0.90 respectively. TO shows notably lower correlations with the Lin and Jiang measures. In contrast, NTO showed reasonably high correlations with all measures, including Lin and Jiang's (Additional file 2). NTO and TO are also highly correlated (rank correlation 0.82, Table 1). Figures 3 and 4 present scatter plots of TO plotted against the six IC-based measures (similar figures can be found for the vector based measures and NTO in Additional file 3 and 4, respectively). The higher correlations of TO with the measures computed using the "maximum" method (Figure 3) than "average" (Figure 4) are evident. We also noted that the Lin and Jiang measures yield a number of gene pairs with similarity values of 1.0 over a wide range of TO values, especially with the "maximum" method (30% of all gene pairs; Figures 3B and 3C). We also present scatter plots for the "average" variants of the IC-based methods to illustrate how they compare against one another (Figure 5).

**Table 1:** Correlation of TO scores with various other similarity measures

	Pearson	Spearman
Resnik	0.56	0.77
Resnik_Max_	0.77	0.87
Lin	0.47	0.74
Lin_Max_	0.64	0.83
Jiang	0.36	0.65
Jiang_Max_	0.58	0.78
NTO	0.65	0.82
Kappa	0.76	0.89
Cosine	0.75	0.82
Weighted Cosine	0.51	0.90

Pearson and rank correlation values were calculated for the scores generated by TO versus each of the seven other measures for the final dataset R_100 K_, containing 100,000 random gene pairs.

**Figure 6:**
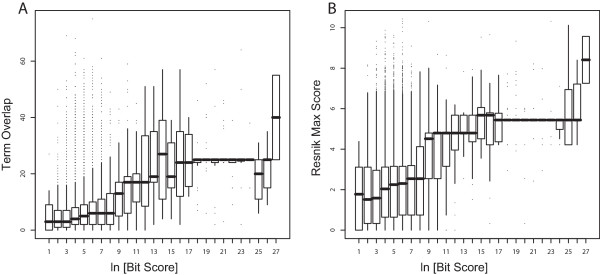
**Comparing sequence and semantic similarity**. A BLAST sequence analysis was performed to calculate a sequence similarity score for each gene pair in the 100 k set for which sequence data was available. Of those gene pairs we considered only the 53,264 which obtained a score greater than zero. Pairs were binned by bit score and the average in each bin plotted against (A) TO scores and (B) Resnik-max scores. Thick horizontal lines indicate medians, boxes indicate interquartile ranges, and whiskers are drawn at 1.5 times the quartile, or the maximum (whichever is closer to the median).

We next considered whether semantic similarity is related to another measure of functional similarity, protein sequence similarity (Figure 6 and Additional file 5). As previously reported, the Resnik-average score was positively rank-correlated with BLAST scores (0.086, p < 10^-16^). Resnik-maximum showed a higher correlation and TO with the highest correlation with BLAST scores (rank correlation 0.125, Table 2). We note that Lord et al. (2003) reported correlations of up to ~0.6. This discrepancy appears to be explained by the binning Lord et al. performed prior to computing correlations.

**Table 2:** Rank correlation of semantic similarity with sequence similarity

TO	NTO	Resnik	Resnik_Max_	Lin	Lin_Max_	Jiang	Jiang_Max_	Kappa	Cosine	Wt.cosine
0.125	0.112	0.086	0.110	0.088	0.104	0.078	0.10	0.106	0.113	0.114

Rank correlation values were calculated for the semantic similarity scores and the sequence similarity bit scores for each gene pair.

**Figure 7:**
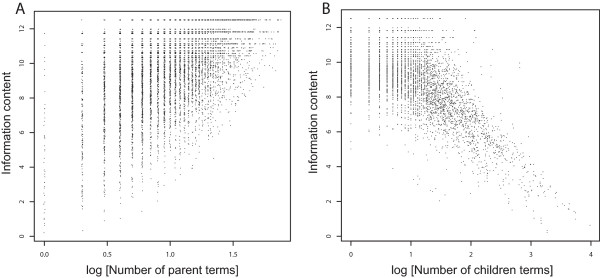
**The relationship between the depth of the GO hierarchy and IC**. For each GO term, we retrieved the total number of parent terms leading back to the root term, and the total number of children terms. Each dot on the plot represents a GO term, for a total of 8424 terms that have been used in at least one gene annotation within the mouse corpus. The log_10 _values are plotted each against the pre-calculated IC for that term; jitter was added to each point to reduce overlapping data.

To help understand the basis for the good agreement between TO/NTO and the information-content measures, we studied the relationship between the location of a term in the GO hierarchy and information content. This is relevant because TO and NTO are fundamentally based on location in the hierarchy. As shown in Figure 7A, the position of a term in the hierarchy is correlated with information content: terms with few parents (near the root of the hierarchy) tend to have low IC (rank correlation 0.26). This is expected because IC increases monotonically as one follows parent-child relationships. The overall good agreement suggests that depth in the hierarchy is a reasonable surrogate for IC. However, the cluster of data points occupying the upper left diagonal of Figure 7A shows that there are many terms near the root with high information content. Figure 7B displays the relationship between IC and the number of child terms (rank correlation 0.68). While the trend is similar to that for the number of parents, there are few cases of terms with many children and high IC. Exceptions can be found, for example "viral reproduction" (GO:0016032) which has 157 children, but an IC of 9.3.

Finally, we examined the running times of the various algorithms on the 100 k dataset (Table 3). TO measure is over 10 times as fast as the other measures. These times exclude the cost of computing the IC values from the corpus (>1 hour, primarily due to the cost of database lookups).

**Table 3:** Running times for some of the similarity measures

	Term Overlap	Normalized Term Overlap	Resnik	Lin	Jiang
Time (s)	226.8	260.5	2979	3092	3165

Time shown is total time taken for the 100,000 gene pair set R100K and excludes the cost of computing the IC values from the corpus. Averaged and maximum variants of the semantic similarity measures show no difference in time, thus only one value is displayed.

## Discussion

In this work we conducted a detailed study of a measure of gene functional similarity based on Gene Ontology terms, Term Overlap. We found that TO compares very well to other semantic similarity measures, and is easier and faster to compute. This suggests that TO can be used as an alternative to the more complex measures that have been proposed. In addition we demonstrate that in general, the various measures are all highly correlated, with some important exceptions. Here we discuss some of the reasons for differences in performance among the methods.

We find that with the IC-based methods, the scores from the TO correlate best with Resnik-maximum and Resnik-average scores. Recent studies have shown that the similarity measure proposed by Resnik out-performs the Lin and Jiang methods in terms of correlation with gene sequence similarities [9,10] and gene expression profiles [23], consistent with our findings. Our sequence analysis findings indicate that TO correlates comparably (even slightly higher) with sequence similarity than Resnik. This suggests that TO is at least as reflective of "true" gene function as the measure used by Lord et al. (2003). However, we point out that overall correlations are low; the difference might be corpus-specific, and there is no unassailable "gold-standard" for evaluating semantic similarity measures.

We found that the Lin and Jiang measures correlate relatively poorly with most of the other methods, while being most similar to NTO. It has been previously shown that the Lin and Jiang methods suffer from what is referred to as the "shallow annotation problem" [7,23]. This is because Lin and Jiang both use the IC of the query genes as well as the LCA. As a result, genes that are annotated at only very shallow levels of the GO hierarchy (e.g., "metabolism") can yield very high semantic similarities. Such pairs are therefore not distinguishable from high-scoring pairs of genes that have "deep" annotations. The effect of shallow annotations can be seen in Figures 3 and 4, where both Lin and Jiang measures have large numbers of points with scores of 1.0, distributed over a wide range of TO scores, including very low TO values. Thus, although the Lin and Jiang methods attempt to capture the nature of the hierarchy in their methods, the effect of the shallow annotation problem shows that these methods can produce misleading results. For example, the gene pair containing Akap1 (A kinase anchor protein 1), and Bbs9 (Bardet-Biedl syndrome 9) using Lin and Jiang methods score a similarity of 1.0. Akap1 is a trans-membrane protein that participates in second messenger signalling [24,25], and has 29 GO terms associated with it (including parents). The function for Bbs9 on the other hand is poorly understood [26] and has only 3 associated terms, including "extracellular space", which it happens to share with Akap1. Despite this weak link, according to both Lin and Jiang methods these genes are not only similar but they generate the maximum attainable score for those measures.

Scrutiny of the data leads us to believe that the NTO scores also suffer from the shallow annotation problem. This is because even if a gene is annotated with only one term, and it shares that term with another gene, the NTO is 1.0. Using the previously mentioned gene pair Akakp1 and Bbs9, the NTO measure generates a high score of 0.75, whereas the TO measure generates a more appropriate low score of 3.0. The Jaccard, Dice and Czekanowski-Dice methods [14,20], which are computed in a similar fashion to NTO but use larger normalizing factors, will ameliorate the shallow annotation problem. However, shallow annotation artifacts will persist when comparing pairs of genes where both have few terms For this reason we favour the raw TO over the normalized overlap measures. On the other hand, the normalized measures have the useful property of yielding values restricted between zero and one.

Others have suggested that using IC values can induce substantial artifacts for terms that are rarely used, but not necessarily very specific [7]. This problem will be particularly acute for organisms with sparse GO annotations. We refer to this problem as the "corpus bias". For example, a high level, general term such as "cell growth" (GO:0016049) should have a significantly high background probability and low IC. However, if the corpus does not contain many genes involved in cell growth, the term will score a low probability and will be incorrectly identified as a high IC specific term. As shown in Figure 7A, most terms near the top of the hierarchy have low IC (Figure 7A). However, there are exceptions, as noted above. These terms located near the top of the hierarchy but with low IC have two potential causes. First, it is possible that depth in the hierarchy is not always related to semantic information content. In other words, there may be terms near the top of the hierarchy that are as "specific" as terms deep in the hierarchy. Second, there may be true corpus bias where terms with many children are rarely used. We can partly distinguish between these possibilities by examining the relationship between IC and the number of child terms (Figure 7B). This showed that high-IC terms almost always have many child terms, arguing against corpus bias. It is still unclear whether the cases of terms with few children and few parents are truly "specific" terms or just parts of the GO which are not yet fully fleshed out. For example, the term "chemoattractant activity" (GO:0042056) is a direct child of the root of the molecular function hierarchy, but has no child terms.

TO appears to avoid the shallow annotation problem. However, it may be questioned whether depth in the hierarchy is a strong enough correlate with IC. TO is in effect based entirely on how many parents a term has, with no consideration of the frequency of use by annotators. Thus two genes annotated with low level terms falling far from the root term, and sharing all parent terms would obtain a high similarity score. On the other hand, if two genes are annotated with high level terms falling closer to the root term, also sharing all parent terms, will obtain a low similarity score. This will work so long as the depth in the hierarchy is a reasonably uniform measure of semantic specificity (in effect, information content). The data in Figure 7A suggest this might not be an entirely safe assumption, but we argue that the overall good behaviour of the overlap statistic argues that the assumption is not completely without basis.

The scores generated using VSM based measures also show a high correlation with TO (>0.8). These methods rely upon a gene-term annotation matrix that essentially flattens the redundant and structured GO terms into a collection of 'independent' terms. The Kappa and Cosine methods weight each of the GO terms equally by using a binary valued matrix and would explain the high correlation with each other, and with TO. On the other hand using weighted values in the matrix delivers scores that still correlate fairly well with TO (higher than the correlation with Resnik) as we found with the Weighted Cosine measure. Both Cosine measures also correlate very well with the NTO measure. This is not surprising, since the dot product of two gene vectors equates to the term overlap, and thus the two measures merely differ by the factor that they normalize the TO value with. This is also in general agreement with results presented recently by Chagoyen et al. (8^th ^Spanish Symposium on Bioinformatics and Computational Biology, 2008).

TO (and NTO) also differs from the other measures in algorithmic complexity. First, computing the IC for each term is an expensive computation, as is the compilation process of the gene-term annotation matrix required for the vector-based methods. The IC requires obtaining counts of each term in GO for all genes; as does the Weighted Cosine method. Making matters worse, in both cases the data generated should in principle be recomputed for the entire database every time the annotations are updated. TO completely avoids this step. In addition, the computation of overlap for a pair of genes is O(N) where N is the number of terms. Computation of the other IC-based measures requires pairwise comparison of all terms for the pair of genes, which is O(N^2^).

In summary, given the generally high correlation among the various measures, it seems reasonable to use the simplest and fastest method when high throughput is necessary. Therefore we expect that TO will be of use for rapidly evaluating algorithms predicting gene functional relationships, and in exploring high-throughput experimental data. For example, in Lee et al. (2004), TO was used to evaluate the performance of an algorithm for predicting gene function on the basis of expression profile similarity. TO is fast enough to use in on-line applications, and is used in the Gemma system  to display gene semantic similarities (Hamer et al., in preparation). Semantic similarity computed by TO could be used to evaluate and examine results of high throughput studies such as yeast 2-hybrid screens or proteomic studies.

## Authors' contributions

PP proposed the overlap measure, and oversaw design and execution of the study. MM implemented algorithms, designed and performed experiments, and performed all of the analysis. PP and MM wrote the manuscript. Both authors read and approved the final manuscript.

## Supplementary Material

Additional File 1 TO correlation scores for multiple sets. Click here for file

Additional File 2 Correlation values amongst various similarity measures. Click here for file

Additional File 3 **TO scores versus scores generated using vector -based measures**. For every gene pair in the 100 k set of gene pairs, the term overlap was calculated and plotted against the scores generated by Cosine, Kappa, and Weighted Cosine measures. Click here for file

Additional File 4 **NTO scores versus TO, Resnik, Lin, and Jiang scores**. For every gene pair in the 100 k set of gene pairs, the normalized term overlap was calculated and plotted against the term overlap scores (A), the averaged variant scores of each of the three semantic similarity measures (B-D), and the maximum variant scores of each of the three semantic similarity measures (E-G). Click here for file

Additional File 5 **Comparing sequence and semantic similarity ("average" variants)**. A BLAST sequence analysis was carried out to calculate a sequence similarity score for each gene pair in the 100 k set for which sequence data was available. Of those gene pairs we considered only the 53,264 which obtained a score greater than zero. Intervals were taken along the x-axis ln [Bit Score] and (A) Resnik, (B) Lin and (C) Jiang scores for the corresponding gene pairs were averaged and plotted. Click here for file
