# Supplementary material for: Gene Ontology term overlap as a measure of gene functional similarity
Source: BMC Bioinformatics. 2008 Aug 4;9:327. doi: 10.1186/1471-2105-9-327 (PMC2518162; doi:10.1186/1471-2105-9-327)
Supplement: Additional File 1 — TO correlation scores for multiple sets. [file 1471-2105-9-327-S1.doc]

**Table A1: TO correlation scores for multiple sets. A)** Pearson correlation values were calculated for the scores generated by the TO versus each of the various semantic similarity metrics and their variants, for four different datasets. The initial test dataset R_test_ contained a total of 1600 gene pairs, whereas R1_10K_ and R2_10K_ datasets contained a total of 10,000 random gene pairs each. **B)** Similarly, the Spearman rank correlation values were calculated for each of the different datasets. Data generated for the final dataset R_100K_, (100,000 random gene pairs) can be found in the main figures).

A) Pearson Correlation Data

| DataSet | Resnik | Resnik_Max_ | Lin | Lin_Max_ | Jiang | Jiang_Max_ |
| --- | --- | --- | --- | --- | --- | --- |
| *Term Overlap* | |  |  |  |  |  |
| R_test_ | 0.58 | 0.80 | 0.42 | 0.52 | 0.25 | 0.50 |
| R1_10K_ | 0.56 | 0.76 | 0.49 | 0.65 | 0.38 | 0.59 |
| R2_10K_ | 0.56 | 0.77 | 0.47 | 0.64 | 0.36 | 0.57 |

**B) Spearman Rank Correlation Data**

| DataSet | Resnik | Resnik_Max_ | Lin | Lin_Max_ | Jiang | Jiang_Max_ |
| --- | --- | --- | --- | --- | --- | --- |
| *Term Overlap* | |  |  |  |  |  |
| R_test_ | 0.63 | 0.84 | 0.50 | 0.68 | 0.39 | 0.66 |
| R1_10K_ | 0.77 | 0.87 | 0.74 | 0.84 | 0.66 | 0.79 |
| R2_10K_ | 0.76 | 0.87 | 0.73 | 0.83 | 0.65 | 0.78 |
