# Supplementary material for: Gene Ontology term overlap as a measure of gene functional similarity
Source: BMC Bioinformatics. 2008 Aug 4;9:327. doi: 10.1186/1471-2105-9-327 (PMC2518162; doi:10.1186/1471-2105-9-327)
Supplement: Additional File 2 — Correlation values amongst various similarity measures. [file 1471-2105-9-327-S2.doc]

**Table A2: Correlation values amongst various similarity metrics.** For the final dataset R_100K_, scores were generated using each of the eleven different similarity metrics. The Pearson correlation (A) and rank correlation (B) of those scores with each other can be found below. Figure 2A and B contain the same data displayed graphically.

A)

|  | Resnik | Resnik_Max_ | Lin | Lin_Max_ | Jiang | Jiang_Max_ | TO | NTO | Cosine | Weighted Cosine | Kappa |
| --- | --- | --- | --- | --- | --- | --- | --- | --- | --- | --- | --- |
| Resnik | 1 |  |  |  |  |  |  |  |  |  |  |
| Resnik_Max_ | 0.83 | 1 |  |  |  |  |  |  |  |  |  |
| Lin | 0.88 | 0.73 | 1 |  |  |  |  |  |  |  |  |
| Lin_Max_ | 0.76 | 0.85 | 0.83 | 1 |  |  |  |  |  |  |  |
| Jiang | 0.68 | 0.58 | 0.93 | 0.75 | 1 |  |  |  |  |  |  |
| Jiang_Max_ | 0.62 | 0.72 | 0.76 | 0.94 | 0.77 | 1 |  |  |  |  |  |
| TO | 0.56 | 0.77 | 0.47 | 0.64 | 0.36 | 0.58 | 1 |  |  |  |  |
| NTO | 0.70 | 0.71 | 0.75 | 0.72 | 0.69 | 0.65 | 0.65 | 1 |  |  |  |
| Cosine | 0.70 | 0.74 | 0.74 | 0.72 | 0.68 | 0.65 | 0.75 | 0.89 | 1 |  |  |
| Weighted Cosine | 0.52 | 0.53 | 0.58 | 0.45 | 0.57 | 0.45 | 0.51 | 0.64 | 0.77 | 1 |  |
| Kappa | 0.66 | 0.72 | 0.70 | 0.68 | 0.63 | 0.62 | 0.76 | 0.81 | 0.98 | 0.50 | 1 |

B)

|  | Resnik | Resnik_Max_ | Lin | Lin_Max_ | Jiang | Jiang_Max_ | TO | NTO | Cosine | Weighted  Cosine | Kappa |
| --- | --- | --- | --- | --- | --- | --- | --- | --- | --- | --- | --- |
| Resnik | 1 |  |  |  |  |  |  |  |  |  |  |
| Resnik_Max_ | 0.91 | 1 |  |  |  |  |  |  |  |  |  |
| Lin | 0.93 | 0.86 | 1 |  |  |  |  |  |  |  |  |
| Lin_Max_ | 0.86 | 0.91 | 0.92 | 1 |  |  |  |  |  |  |  |
| Jiang | 0.73 | 0.72 | 0.90 | 0.87 | 1 |  |  |  |  |  |  |
| Jiang_Max_ | 0.74 | 0.81 | 0.86 | 0.95 | 0.92 | 1 |  |  |  |  |  |
| TO | 0.77 | 0.87 | 0.74 | 0.83 | 0.65 | 0.78 | 1 |  |  |  |  |
| NTO | 0.79 | 0.80 | 0.82 | 0.81 | 0.77 | 0.77 | 0.82 | 1 |  |  |  |
| Cosine | 0.81 | 0.83 | 0.83 | 0.83 | 0.78 | 0.79 | 0.89 | 0.94 | 1 |  |  |
| Weighted Cosine | 0.86 | 0.89 | 0.88 | 0.87 | 0.78 | 0.80 | 0.82 | 0.88 | 0.90 | 1 |  |
| Kappa | 0.78 | 0.82 | 0.80 | 0.81 | 0.75 | 0.77 | 0.90 | 0.88 | 0.98 | 0.87 | 1 |
