# Supplementary material for: Gene Ontology term overlap as a measure of gene functional similarity
Source: BMC Bioinformatics. 2008 Aug 4;9:327. doi: 10.1186/1471-2105-9-327 (PMC2518162; doi:10.1186/1471-2105-9-327)
Supplement: Additional File 3 — TO scores versus scores generated using vector -based measures. For every gene pair in the 100 k set of gene pairs, the term overlap was calculated and plotted against the scores generated by Cosine, Kappa, and Weighted Cosine measures. [file 1471-2105-9-327-S3.doc]

| **A)** | **B)** |
| --- | --- |
| **C)** | |

**Additional file 3: Relationship between Term Overlap and alternate methods A) Cosine B) Kappa Statistics C) Weighted Cosine.**
