# Supplementary material for: Gene Ontology term overlap as a measure of gene functional similarity
Source: BMC Bioinformatics. 2008 Aug 4;9:327. doi: 10.1186/1471-2105-9-327 (PMC2518162; doi:10.1186/1471-2105-9-327)
Supplement: Additional File 4 — NTO scores versus TO, Resnik, Lin, and Jiang scores. For every gene pair in the 100 k set of gene pairs, the normalized term overlap was calculated and plotted against the term overlap scores (A), the averaged variant scores of each of the three semantic similarity measures (B-D), and the maximum variant scores of each of the three semantic similarity measures (E-G). [file 1471-2105-9-327-S4.doc]

| **A)** | **B)** |
| --- | --- |
| **C)** | **D)** |
| **E)** | **F)** |
| **G)** |  |

**Additional file 4: NTO scores versus TO, Resnik, Lin, and Jiang scores.**
