# Supplementary material for: Gene Ontology term overlap as a measure of gene functional similarity
Source: BMC Bioinformatics. 2008 Aug 4;9:327. doi: 10.1186/1471-2105-9-327 (PMC2518162; doi:10.1186/1471-2105-9-327)
Supplement: Additional File 5 — Comparing sequence and semantic similarity ("average" variants). A BLAST sequence analysis was carried out to calculate a sequence similarity score for each gene pair in the 100 k set for which sequence data was available. Of those gene pairs we considered only the 53,264 which obtained a score greater than zero. Intervals were taken along the x-axis ln [Bit Score] and (A) Resnik, (B) Lin and (C) Jiang scores for the corresponding gene pairs were averaged and plotted. [file 1471-2105-9-327-S5.doc]

| **A)** | **B)** |
| --- | --- |
| **C)** | |

**Additional file 5: Comparing sequence and semantic similarity. (“average” variants).**
